# Atmospheric undular bores

**DOI:** 10.1007/s00208-023-02624-8

**Published:** 2023-04-29

**Authors:** A. Constantin, R. S. Johnson

**Affiliations:** 1 Faculty of Mathematics, University of Vienna, Oskar-Morgenstern-Platz 1, 1090 Vienna, Austria; 2 School of Mathematics, Statistics and Physics, Newcastle University, Newcastle upon Tyne, NE1 7RU UK

**Keywords:** 86A10, 35C07, 34C15

## Abstract

We show that a recently-derived model for the propagation of nonlinear waves in the atmosphere admits undular bores as travelling-wave solutions. These solutions represent waves consisting of a damped oscillation behind a front that is preceded by a uniform breeze-type flow. The generation of such wave profiles requires a jump in the heat source across the leading front of the wave, a feature that is consistent with observations.

## Introduction

**Fig. 1:**
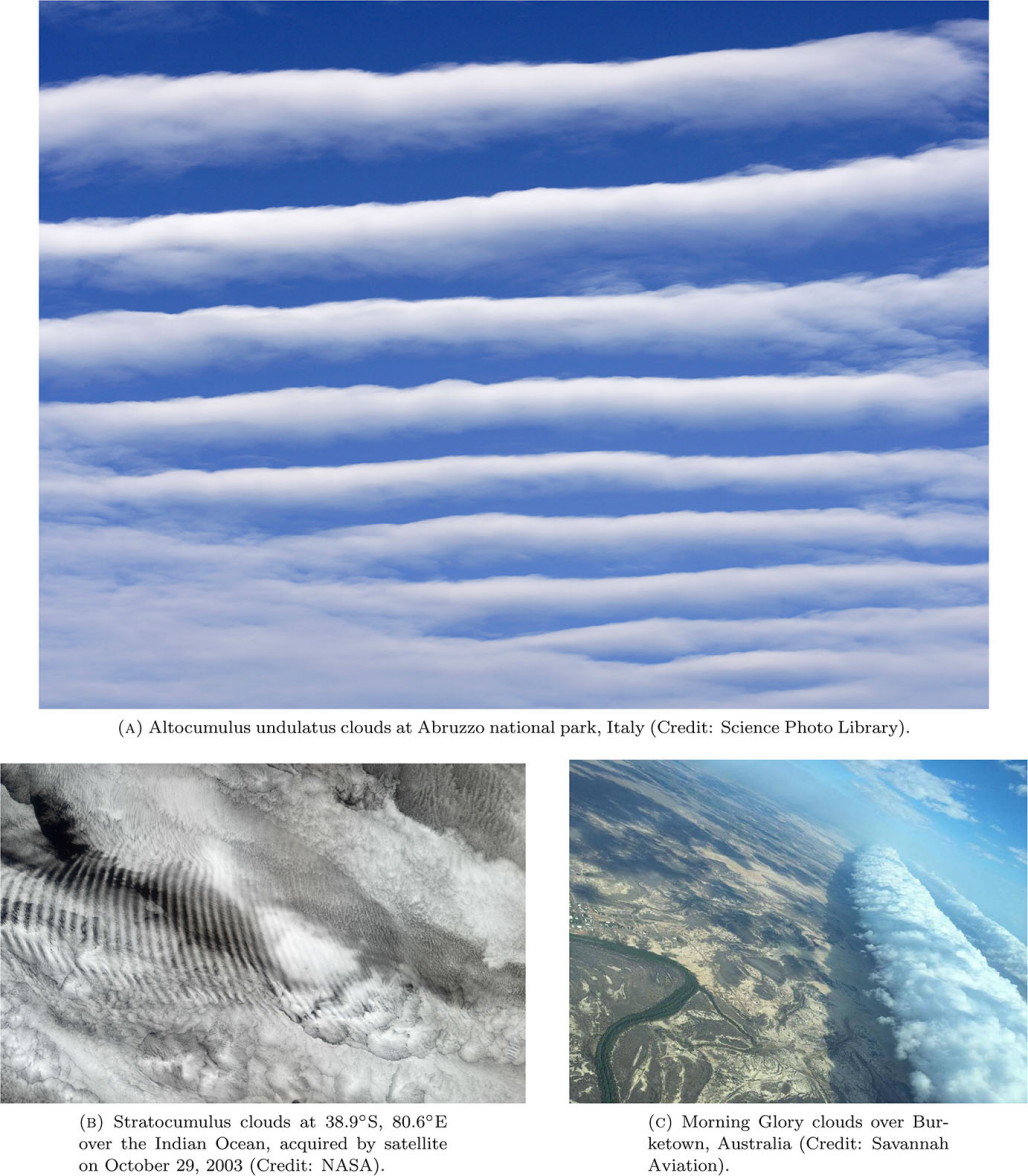
Photographs of some atmospheric undular bores

The morning glory wave-structure is a quite amazing sight, but producing a robust and consistent theory for its investigation is a considerable challenge. This phenomenon comprises a travelling wave which propagates with little perturbation of the background uniform shear flow (a breeze that typically varies only with height), followed by a steep, rising front with trailing, damped oscillations. The result is a spectacular meteorological undular bore which often becomes visible during the early morning as bands of clouds, sometimes stretching from horizon to horizon (see Fig. 1). An important element in the production of these waves is the thermal inversion (the reversal of the normal tropospheric temperature reduction with height – see [6]), where a layer of cooler air is overlain by a layer of warmer air. This occurs typically after a warm airflow encounters colder, humid air. The warmer air is lifted and glides over the cold air-mass, thereby producing the thermal inversion. Then, as a wave crest approaches, the parcels of humid air rise and cool, condensation occurs and clouds are formed. Subsequently, as the wave crest passes, the parcels of air descend and warm up, so that the cloud evaporates. The clouds usually disappear by mid-morning, as the overall air temperature rises. It should be emphasised that, in this process, the clouds are not carried along with the air flow; rather, clouds are continuously being formed at the leading edge of each wave and continuously eroded at the trailing edge. Atmospheric undular bores often propagate at speeds in excess of 10 m/s and may last for hours (see [11]). More details of the background to this phenomenon, and to the underlying physics, can be found in [8].

As we have indicated, the mathematical description of these atmospheric undular bores is far from routine work. It is a fairly straightforward exercise to write down model equations that appear to possess the properties observed in the morning glory; see, for example, [4, 12, 18]. Some of the attempts at modelling involve arguing that a direct connection exists between these waves and the Korteweg-de Vries or Benjamin-Ono equations; see [2]. Typically, the time and length scales in these model equations are not quite correct but, by analogy, it is argued that something along these lines is the appropriate starting point. The application to the morning glory waves then requires the scales and available coefficients to be matched to the data in order to recover what is observed; see, for example, [14, 22]. Of course, numerical simulations provide some useful clues, as does the application of arguments that draw an analogy with the undular bores observed in rivers. We refer to [15, 16] for a discussion of hydraulic bores but we note, even in this simpler context, that it is difficult to construct closed-form representations of the wave profiles. Moreover, whereas hydraulic bores in water are typically generated by fast tidal currents being funnelled into narrow estuaries (see [1] for some beautiful photographs of tidal bores on the Severn river), atmospheric bores are formed by warm air flowing over colder air. Also, in stark contrast to water flows, which typically transport energy but not mass (see the discussion in [5]), atmospheric bores transport air and energy (see [19]). For example, the lifting of the air parcels by the passage of the bore, with nett vertical displacements of about 1 km, plays an important rôle in the nocturnal convection over the Great Plains in the central US (see [11]), which produces most of the warm-season precipitation [17]. While high humidity makes the magnificent morning glory clouds over the Gulf of Carpentaria in Australia clearly visible (see [20]), bores over the central US are typically traced by means of radar images (partly because they often occur during the night, when direct confirmation by an observer is difficult and satellite imagery is not available).

In all the material cited above, and in the many others recorded in [8], there is no careful and comprehensive derivation of a suitable equation (or system) from the governing equations that describe the fluid dynamics of the atmosphere. That this is possible is demonstrated in detail in [8]. (We provide a brief overview of the relevant derivation, so that the reader is able to put the results of the current work directly in context.) A number of special solutions were highlighted in this earlier paper, each extracting an important property of these wave systems, but very little more was offered. The work presented here attempts to combine all the salient features of the morning glory in one solution of this model equation. As we shall see, this new solution captures all the properties of atmospheric undular bores.

The plan for this paper is as follows. After presenting in Sect. 2 a brief outline of the derivation of the equation which describes nonlinear wave propagation in the troposphere, we show in Sect. 3 how a Liénard-type second-order ordinary differential equation underlies the modelling of undular bores. In Sect. 4 we investigate the qualitative behaviour of the solutions of the Liénard equation; this provides a firm basis on which to present a discussion of the wave structure that is typical of the undular bores. Finally, in Sect. 5, we combine all these results to present a detailed mathematical description of the morning glory, which includes the rôle of the background atmosphere and associated breezes, as well as a numerical simulation that depicts a typical undular bore.

## Preliminaries

We rely on a recently-derived model for the nonlinear propagation of atmospheric waves (see [8]), derived from the governing equations for flows in the troposphere, written in rotating spherical coordinates – the Navier–Stokes equation with variable eddy viscosities, the equation of mass conservation for a compressible fluid, the equation of state for the atmosphere and the first law of thermodynamics – using only the thin-shell approximation. The model accommodates waves propagating horizontally through the atmosphere in any direction, with the underlying asymptotic structure requiring that the length scale of the wave packet should be the same as the thickness scale of the troposphere (about 16 km), with the length scale of the wave front based on the radius of the Earth. Thus the wave packet might extend tens of kilometres, but the wave front could be a thousand kilometres long. The time scale of the motion was given by $1/\varOmega ' \approx 3\,\tfrac{1}{2}$ hours, where $\varOmega '$ is the constant angular speed of rotation of the Earth.

The spherical coordinates $(\varphi ,\theta ,r')$ in the rotating frame are the distance $r'$ from the centre, the angle of latitude $\theta \in [-\tfrac{\pi }{2},\tfrac{\pi }{2}]$ and the angle of longitude $\varphi \in [0,2\pi )$. They are redefined as

$$\begin{aligned} \varphi -\varphi _0=\Phi \cos \alpha - \varepsilon \,\Theta \sin \alpha ,\qquad \theta -\theta _0=\Phi \sin \alpha + \varepsilon \,\Theta \cos \alpha , \end{aligned}$$

for a clockwise rotation of the coordinates about the fixed point $(\varphi _0,\theta _0)$ through the fixed angle $\alpha$; here $\varepsilon =H'/R'$ is the thin-shell parameter, with $H'$ the maximum thickness of the troposphere and $R'$ an average radius of the Earth. Throughout, the prime denotes dimensional (physical) variables so, in particular, we write the time variable as $t'=t/\varOmega '$ and the radial variable as $r'=R'(1+\varepsilon Z)$, where *t* and *Z* are nondimensional variables. Further, the horizontal velocity components, $(u',\,v')$, in this spherical system, are rotated to produce components that are in the direction of propagation ($V'$) and along the wave front ($U'$):

$$\begin{aligned} u' = U' \cos \alpha - V' \sin \alpha ,\quad v' = U'\sin \alpha + V' \cos \alpha ; \end{aligned}$$

the vertical component, $w'$, is unchanged; we use $H'\varOmega ' \approx 1.2$ m/s as the speed scale. Further details of the non-dimensionalisation are given in [8], and hereafter nondimensional variables are written without the prime. The process then involves the perturbation of a general background state of the atmosphere, eventually producing a nonlinear equation for the stream function, $\Psi$, which describes the leading-order approximation, as $\varepsilon \rightarrow 0$, of the velocity components $(V_0,\,w_0)$ with the leading-order component along the wave front taken as $U_0=0$. The evolution equation for $\Psi (\Phi ,\Theta ,Z,t)$, at leading order, is

2.1 $$\begin{aligned}&\frac{\partial ^2\Psi }{\partial Z \partial t} + \frac{1}{\rho _0} \,\frac{\partial \Psi }{\partial Z} \, \frac{\partial ^2\Psi }{\partial Z \partial \Theta } - \frac{\partial \Psi }{\partial \Theta }\,\frac{\partial }{\partial Z}\Big ( \frac{1}{\rho _0}\,\frac{\partial \Psi }{\partial Z} \Big ) - \sigma \Big ( S\, \frac{\partial \Psi }{\partial Z} + C \cos \alpha \,\frac{\partial \Psi }{\partial \Theta } \Big ) \nonumber \\&\qquad - \frac{1}{R_e}\,\Big \{ \frac{\partial }{\partial Z} \Big ( \mu _v\,\frac{\partial }{\partial Z}\Big ) +\mu _h\,\Big ( \cos ^2\alpha + \frac{\sin ^2\alpha }{C}\Big )\,\frac{\partial ^2}{\partial \Theta ^2}\Big \} \Big ( \frac{1}{\rho _0}\,\frac{\partial \Psi }{\partial Z} \Big )= K(\Phi ,Z,t)\,,\nonumber \\ \end{aligned}$$

where $\rho _0(\xi )$ with $\xi ={\mathfrak {g}}Z + \frac{1}{2}\,S^2$ is the background density in the atmosphere, and

$$\begin{aligned}{} & {} {\mathfrak {g}}=\frac{g'H'}{\varOmega '^2R'^2},\quad R_e=\frac{\overline{\rho }'\varOmega 'H'^2}{\overline{\mu }'} ,\quad S=\sin (\theta _0 + \Phi \sin \alpha ),\quad C=\cos (\theta _0 + \Phi \sin \alpha ),\\{} & {} \quad \sigma = \frac{2(\sin ^2\alpha + C\cos ^2\alpha )}{(1-C)\sin \alpha \cos \alpha }. \end{aligned}$$

with $g' \approx 9.8$ m/s^2^ the average acceleration of gravity at the surface of the Earth. We have also introduced: an average density of the atmosphere $\overline{\rho }'$ and an average dynamic eddy viscosity $\overline{\mu }'$, with $\overline{\mu }'\mu _v(Z)$ and $\overline{\mu }'\mu _h(Z)$ the vertical and horizontal dynamic eddy viscosities, respectively. The Reynolds number, $R_e$, has been treated as $\textrm{O}(1)$ under the limiting process $\varepsilon \rightarrow 0$, so that the dominant viscous effects are retained. The forcing term, $K(\Phi ,Z,t)$, is any appropriate external heat-forcing that drives and maintains the motion. We note, in passing, that any variation along the wave front, which depends on $\Phi$, appears only parametrically in this equation. Finally, we record the velocity components expressed in terms of the stream function:

2.2 $$\begin{aligned} V_0= \frac{1}{\Big ( \cos ^2\alpha + \frac{\sin ^2\alpha }{C}\Big )\rho _0}\, \frac{\partial \Psi }{\partial Z} \,,\qquad w_0= -\,\frac{1}{\rho _0}\,\frac{\partial \Psi }{\partial \Theta }\,. \end{aligned}$$

Of special interest are travelling-wave solutions that describe wave propagation for fixed $\Phi$, at constant speed $c(\Phi )$, without change of shape. These solutions correspond to stream functions of the form

$$\begin{aligned} \Psi (\Phi , \Theta ,Z,t)=\widehat{\Psi }(\Phi ,\,\widehat{\Theta },\,\widehat{Z}) \quad \text {with}\quad \widehat{\Theta }=\Theta - c(\Phi )t,\quad \widehat{Z}=\big (\cos ^2\alpha + \tfrac{\sin ^2\alpha }{C}\big )\,Z, \end{aligned}$$

and associated velocity components

2.3 $$\begin{aligned} \widehat{V}_0= \frac{1}{\rho _0(\xi )}\, \frac{\partial \widehat{\Psi }}{\partial \widehat{Z}} \,,\qquad \widehat{w}_0= -\,\frac{1}{\rho _0(\xi )}\,\frac{\partial \widehat{\Psi }}{\partial \widehat{\Theta }}\,. \end{aligned}$$

In the two-dimensional frame of reference moving horizontally at the wave speed $c(\Phi )$, the particle paths $t \mapsto (\Phi ,\,\widehat{\Theta }(t),\,\widehat{Z}(t))$, obtained by solving the system

2.4 $$\begin{aligned} {\left\{ \begin{array}{ll} \displaystyle \frac{\textrm{d}\widehat{\Theta }}{\textrm{d}t} =\widehat{V_0}(\Phi ,\,\widehat{\Theta }(t),\,\widehat{Z}(t))\,,\\[0.33cm] \displaystyle \frac{\textrm{d}\widehat{Z}}{\textrm{d}t}=\widehat{w}_0(\Phi ,\,\widehat{\Theta }(t),\,\widehat{Z}(t))\,, \end{array}\right. } \end{aligned}$$

(since $U_0=0$ ensures that $\Phi$ remains constant for each individual particle), coincide with the level sets of the function $\widehat{\Psi }$ because (2.3) and (2.4) yield

$$\begin{aligned} \frac{\textrm{d}}{\textrm{d}t}\, \widehat{\Psi }(\Phi ,\,\widehat{\Theta }(t),\,\widehat{Z}(t))=0. \end{aligned}$$

This shows that the profile of the stream function $\Psi$, at fixed $\Phi$, offers insight into the global dynamics of these atmospheric flows.

A simplification of the explicit appearance of the density $\rho _0$ in Eq. (2.1) can be obtained by introducing

2.5 $$\begin{aligned} z=\int _0^Z \rho _0\big ( gZ' + \tfrac{1}{2}\,S^2\big )\,\textrm{d}Z'\,. \end{aligned}$$

This is valid for any general form of the density throughout the troposphere, and the positivity of the density ensures that we may treat $\rho _0=d(y,z)$, in terms of the new variables (*x*, *y*, *z*) with $(\Theta ,\,\Phi )=(x,y)$. Setting $\Psi =\psi (x,y,z,t)$, Eq. (2.1) becomes

2.6 $$\begin{aligned} \frac{\partial ^2\psi }{\partial z \partial t} + \frac{\partial \psi }{\partial z} \, \frac{\partial ^2\psi }{\partial z \partial x} - \frac{\partial \psi }{\partial x}\,\frac{\partial ^2 \psi }{\partial z^2} - \beta \, \frac{\partial \psi }{\partial z} - \gamma \,\frac{\partial \psi }{\partial x} - \Big \{ \frac{\partial }{\partial z} \Big ( m\,\frac{\partial }{\partial z}\Big ) +M\,\frac{\partial ^2}{\partial x^2}\Big \}\,\frac{\partial \psi }{\partial z} = k(y,z,t)\,, \end{aligned}$$

where

$$\begin{aligned}{} & {} \beta (y)=\sigma S,\qquad \gamma (y,z)=\frac{\sigma C \cos \alpha }{d} ,\qquad m(y,z)=\frac{\rho _0 \mu _v}{R_e},\\{} & {} \quad M(y,z)=\frac{\mu _h}{\rho _0 R_e}\,\Big (\cos ^2\alpha + \frac{\sin ^2\alpha }{C}\Big ),\qquad k(y,z,t) = \frac{K}{\rho _0}. \end{aligned}$$

Correspondingly, the velocity components (2.2) can be written as

2.7 $$\begin{aligned} V_0= \frac{C}{C\cos ^2\alpha + \sin ^2\alpha }\, \frac{\partial \psi }{\partial z} \,,\qquad w_0= -\,\frac{1}{d}\,\frac{\partial \psi }{\partial x}\,. \end{aligned}$$

Three important types of solution to Eq. (2.6) were obtained in [8], for special choices of the heat forcing *k*, and of the vertical and horizontal eddy viscosities *m* and *M*:

- Breeze-like horizontal flows (uniform in the *x*-variable at every fixed height), described by stream functions of the form $\psi (z,t)$. Such flows are typically initiated by the different solar heating of land and water surfaces, with marine air advancing onto land as a sea breeze (see [23]).
- Shallow-water bore-like solutions, described by stream functions of the form
  $$\begin{aligned} \psi (x,y,z,t)= a(y,z) + b(y,z) \,\{1- \chi \, \tanh (\kappa [x-ct])\} \end{aligned}$$
  where $\chi >0$, $\kappa >0$, $c>0$ depend only on *y*, representing smooth travelling-wave fronts that bring about the transition between the levels $b(y,z) \{1+ \chi \}$ for $x \rightarrow -\infty$ and $b(y,z) \{1- \chi \}$ for $x \rightarrow \infty$ (relative to *a*). This simple model of a bore is commonly used in shallow-water theory – a framework unable to predict undulations because the vertical component of the velocity is neglected (see the discussion in [9]). Note that the vertical velocity component in an atmospheric undular bore often exceeds 2 m/s (see the data in [11]).
- Uniformly oscillating solutions, described by stream functions of the form
  $$\begin{aligned} \psi (x,y,z,t)= a(y,z) + \chi \,\textrm{e}^{\kappa z} \cos (\kappa [x-ct]) \end{aligned}$$
  where $\chi \ne 0$, $\kappa \ne 0$, $c \ne 0$ depend only on *y*. Such motions are typically observed as adiabatic buoyancy oscillations in a stably stratified atmosphere and are also captured by linear theory (see [7]): for average tropospheric conditions the period of such buoyancy oscillations is about 8 min [13]. However, damped oscillations are a hallmark of non-turbulent atmospheric bores (see the discussion in [24]).

In this paper we present a solution which essentially combines all these elements (a breeze-like flow as a precursor of a bore, with the jump followed by damped oscillations) and so confirms the relevance of Eq. (2.1) to the modelling of undular-bore phenomena in the atmosphere. Our approach goes beyond the usual analyses that use, piecemeal, different models to describe different components of the atmospheric undular bore. We show that this new travelling-wave solution captures all the salient features of phenomena such as the morning glory: damped oscillations behind the leading front, with a horizontally uniform flow ahead and a wave structure generated by a jump in the heat source across the front – a feature consistent with observations.

## Travelling-wave solutions with damped oscillations

The over-arching assumption that we make here is that the solution of interest is expressed as a height-dependent travelling wave in the form

3.1 $$\begin{aligned} \psi = a(y,z) + b(y,z)\, F(x-ct, y)\,. \end{aligned}$$

where *c* is the constant speed of propagation; the parametric dependence on *y* has been included, but it will play no rôle in the development, so we will suppress it hereafter. (It could be reinstated and used, for example, to limit the extent of the wave front in the *y*-direction.) For (3.1) to be a solution of (2.6) requires

3.2 $$\begin{aligned}&-c\,\displaystyle \frac{\textrm{d}b}{\textrm{d} z} + \frac{\textrm{d}a}{\textrm{d} z}\,\frac{\textrm{d}b}{\textrm{d} z} - b\,\frac{\textrm{d}^2 a}{\textrm{d} z^2} - \gamma \,b= p\,\Big \{ \Big (\frac{\textrm{d}b}{\textrm{d} z}\Big )^2 - b\,\frac{\textrm{d}^2b}{\textrm{d} z^2}\Big \}\,, \end{aligned}$$

3.3 $$\begin{aligned}&\beta \,\displaystyle \frac{\textrm{d}b}{\textrm{d} z} + \frac{\textrm{d}}{\textrm{d} z}\,\Big \{m\,\frac{\textrm{d}^2b}{\textrm{d} z^2} \Big \}= q\,\Big \{ \Big (\frac{\textrm{d}b}{\textrm{d} z}\Big )^2 - b\,\frac{\textrm{d}^2b}{\textrm{d} z^2}\Big \}\,, \end{aligned}$$

3.4 $$\begin{aligned}&M\,\displaystyle \frac{\textrm{d}b}{\textrm{d} z} = r\,\Big \{ \Big (\frac{\textrm{d}b}{\textrm{d} z}\Big )^2 - b\,\frac{\textrm{d}^2b}{\textrm{d} z^2}\Big \}\,, \end{aligned}$$

where *p*, *q* and *r* are constants. These three equations are in the two unknowns, *a* and *b*; we must therefore accept that we shall have to make special choices for *m* and/or *M* in order to complete the solution. We anticipate, however, provided that we retain some appropriate form of the variable viscosities, that the fine detail of how the viscosities vary will not significantly alter the main structure of the solution. The resulting equation for $F(x-ct)$ now becomes

3.5 $$\begin{aligned} p\,\frac{\textrm{d} F}{\textrm{d} \xi } + F\,\frac{\textrm{d} F}{\textrm{d} \xi } - q\,F - r\,\frac{\textrm{d}^2F}{\textrm{d} \xi ^2}=\frac{k + \beta \,\displaystyle \frac{\textrm{d}a}{\textrm{d} z} + \displaystyle \frac{\textrm{d}}{\textrm{d} z}\,\Big \{m\,\frac{\textrm{d}^2 a}{\textrm{d} z^2} \Big \}}{\Big (\displaystyle \frac{\textrm{d}b}{\textrm{d} z}\Big )^2 - b\,\frac{\textrm{d}^2b}{\textrm{d} z^2}}=k_0\,, \end{aligned}$$

where $\xi =x-ct$ and $k_0$ is a constant. Note that:

- Setting $p=0$ in (3.5) leads to no simplification, since the new form of (3.5) can be brought back to the original version by adding suitable constants to *F* and $k_0$.
- For $q=r=0$ Eq. (3.5) simplifies to the separable equation
  $$\begin{aligned} (p+F)\,\frac{\textrm{d} F}{\textrm{d} \xi } = k_0. \end{aligned}$$
- For $r=0$ and $q \ne 0$ Eq. (3.5) simplifies to
  $$\begin{aligned} p\,\frac{\textrm{d} F}{\textrm{d} \xi } + F\,\frac{\textrm{d} F}{\textrm{d} \xi } - q\,F = k_0, \end{aligned}$$
  with general solution given implicitly by
  $$\begin{aligned} q(\xi + \tilde{\xi })= F( q\xi ) - \Big ( \frac{k_0}{q} - p\Big )\,\ln \Big | F(q\xi ) + \frac{k_0}{q}\Big | \quad \text {for some}\quad \tilde{\xi } \in {\mathbb R}. \end{aligned}$$
- For $q=0$ and $r \ne 0$ Eq. (3.5) simplifies to
  $$\begin{aligned} p\,\frac{\textrm{d} F}{\textrm{d} \xi } + F\,\frac{\textrm{d} F}{\textrm{d} \xi } - r\,\frac{\textrm{d}^2F}{\textrm{d} \xi ^2} = k_0, \end{aligned}$$
  transformed by integration to the Riccati equation
  $$\begin{aligned} \frac{p}{r}\,F + \frac{1}{2r}\,F^2 - \frac{k_0}{r}\,\xi + \tilde{k} = \frac{\textrm{d} F}{\textrm{d} \xi }, \end{aligned}$$
  for some $\tilde{k} \in {\mathbb R}$. The substitution $F=-2r\,\varphi '/\varphi$ transforms it into the linear second-order differential equation
  3.6 $$\begin{aligned} \frac{\textrm{d}^2 \varphi }{\textrm{d} \xi ^2} - 2p\,\frac{\textrm{d} \varphi }{\textrm{d} \xi } + \Big ( \tilde{k} - \frac{k_0}{r}\,\xi \Big )\,\varphi =0\,. \end{aligned}$$
  For $k_0=0$ the general solution of the corresponding constant-coefficient equation is
  $$\begin{aligned} \varphi (\xi )={\left\{ \begin{array}{ll} \textrm{e}^{p\xi } \Big \{ \varphi _1\, \textrm{e}^{\xi \sqrt{p^2-\tilde{k}}} + \varphi _2\, \textrm{e}^{-\xi \sqrt{p^2-\tilde{k}}}\Big \} \quad \text {if}\quad \tilde{k} < p^2,\\ \textrm{e}^{p\xi } \Big \{ \varphi _1\, \sin \Big (\xi \sqrt{\smash [b]{\tilde{k}-p^2}}\Big ) + \varphi _2\, \cos \Big (\xi \sqrt{\smash [b]{\tilde{k}-p^2}}\Big )\Big \} \quad \text {if}\quad \tilde{k} > p^2,\\ \textrm{e}^{p\xi } ( \varphi _1\,\xi + C_2)\quad \text {if}\quad \tilde{k} = p^2, \end{array}\right. } \end{aligned}$$
  for arbitrary constants $\varphi _1,\,\varphi _2 \in {\mathbb R}$. For $k_0 \ne 0$ the general solution is given by (see [21])
  $$\begin{aligned} \varphi (\xi )=\textrm{e}^{p\xi }\,\sqrt{\xi }\,\Big \{ \varphi _1 J_{1/3}\Big ( \tfrac{2}{3}\,\sqrt{\smash [b]{-\tfrac{k_0}{r}}\,\xi ^{3/2}}\Big ) + \varphi _2 Y_{1/3}\Big ( \tfrac{2}{3}\,\sqrt{\smash [b]{-\tfrac{k_0}{r}}\,\xi ^{3/2}}\Big )\Big \} \end{aligned}$$
  where $J_{1/3}$ and $Y_{1/3}$ are the Bessel functions of the first and second kind, while $\varphi _1,\,\varphi _2 \in {\mathbb R}$ are arbitrary constants. Since for a non-trivial solution $\varphi$ of (3.6) the functions $\varphi$ and $\varphi '$ cannot vanish simultaneously, we see that the above oscillatory solutions to (3.6) correspond to solutions of (3.5) that blow-up.

The previous discussion shows that for $q=0$ and $r=0$ equation (3.5) does not admit oscillatory solutions.

For $qr \ne 0$ it is convenient to normalise Eq. (3.5) by writing

$$\begin{aligned} F=F_0 \,f,\qquad \xi = \xi _0\,X, \end{aligned}$$

where $F_0 \ne 0$ and $\xi _0 >0$ are constants; we then choose

$$\begin{aligned} F_0=-\frac{r}{\xi _0} ,\quad \xi _0= \sqrt{\Big | \frac{r}{q}\Big |} \end{aligned}$$

and write

$$\begin{aligned} \frac{p}{F_0}=\nu ,\quad -\frac{rk_0}{F_0^3}=\kappa , \end{aligned}$$

to give

3.7 $$\begin{aligned} \frac{\textrm{d}^2 f}{\textrm{d} X^2} + f\,\,\frac{\textrm{d} f}{\textrm{d} X} + \text {sgn}(rq) \,f+ \nu \,\frac{\textrm{d} f}{\textrm{d} X}= \kappa \,. \end{aligned}$$

Under these transformations, the velocity components become

3.8 $$\begin{aligned} V_0= \frac{C}{C\cos ^2\alpha + \sin ^2\alpha }\,\Big ( \frac{\textrm{d}a}{\textrm{d} z} + F_0\,\frac{\textrm{d} b}{\textrm{d} z}\,f\Big ) \,,\qquad w_0= -\,\frac{F_0\,b(z)}{\xi _0 \,d(y,z)}\,\frac{\textrm{d} f}{\textrm{d} X}\,. \end{aligned}$$

Before we embark on the construction of a solution of Eqs. (3.2)-(3.4) and (3.7), we make a few general observations about Eq. (3.7), which is the equation at the heart of the analysis. This equation looks disarmingly simple, but it is not! To see this, we transform according to

$$\begin{aligned} \frac{\textrm{d} f}{\textrm{d} X}=G\Big ( \frac{(f+ \nu )^2}{2}\Big ), \end{aligned}$$

which gives the Abel equation of the second kind

3.9 $$\begin{aligned} G\,\frac{\textrm{d} G}{\textrm{d} Y} + G = \pm \frac{\kappa + \nu \,\text {sgn}(rq)}{\sqrt{2Y}} - \text {sgn}(rq) \,, \end{aligned}$$

where $Y=\tfrac{1}{2}\,(f + \nu )^2$ and the choice of ± in (3.9) is given by the sign of $(f + \nu )$. The fact that the forcing term in (3.9) combines a constant with a term proportional to $1/\sqrt{Y}$ immediately precludes any possibility of finding a solution in closed form, unless $\kappa + \nu \,\text {sgn}(rq)=0$ (see the discussion in [21]). For $\kappa + \nu \,\text {sgn}(rq)=0$ we can proceed, as (3.9) simplifies to

$$\begin{aligned} G\,\frac{\textrm{d} G}{\textrm{d} Y} + G = \pm 1, \end{aligned}$$

but this is not a relevant choice for solutions of interest here – there are no oscillatory solutions. Despite the non-availability of explicit solutions for $\kappa + \nu \,\text {sgn}(rq) \ne 0$, we are able to make a simple observation which allows us to make some headway in this case. We seek a solution *f* to (3.7) which approaches a constant $f_0$ as $X \rightarrow - \infty$ and which oscillates (this being an appropriate behaviour for the upper level in an undular bore); to do this we set

$$\begin{aligned} f \sim f_0 + s\,\textrm{e}^{\lambda X}, \end{aligned}$$

where $f_0$, *s* and $\lambda$ are constants, with $\mathfrak {Re} (\lambda )>0$ and $\mathfrak {Im} (\lambda ) \ne 0$. This is a suitable asymptotic solution of Eq. (3.7) when we have

$$\begin{aligned} f_0=\text {sgn}(rq)\,\kappa \quad \text {and}\quad \lambda = - \frac{f_0+\nu }{2} \pm \sqrt{\frac{1}{4}\,(f_0+\nu )^2 - \text {sgn}(rq)}, \end{aligned}$$

and then the solution that we seek requires

3.10 $$\begin{aligned} \text {sgn}(rq) =1 \quad \text {and}\quad -2< f_0+\nu< 0 \quad (\text {or}\ -2< \kappa + \nu <0) \,. \end{aligned}$$

This asymptotic result can be embedded within a more rigorous analysis of the dynamics of the equation

3.11 $$\begin{aligned} \frac{\textrm{d}^2 \widehat{f}}{\textrm{d} X^2} + \widehat{f}\,\frac{\textrm{d} \widehat{f}}{\textrm{d} X} + \widehat{f} - \eta \,\frac{\textrm{d} \widehat{f}}{\textrm{d} X}= 0\,, \end{aligned}$$

as we show in the next section. Eq. (3.11) is obtained by taking $\text {sgn}(rq) =1$ (as we found above) and setting $\widehat{f}=f -\kappa$ in (3.7), with $\eta =-(\kappa + \nu ) \in (0,2)$.

## Global dynamics of the Liénard equation

We now discuss the global dynamics of the autonomous Liénard Eq. (3.11) with parameter $\eta \in (1,2)$. There is a vast research literature devoted to qualitative studies of Liénard type equations (see the discussion in [25]) but mainly from the perspective of limit cycles; the specific form (3.11) is not encountered in these considerations. Equation (3.11) looks disarmingly simple, but, as discussed in Sect. 3, there are no explicit non-trivial solutions available. We prove the following result.

### Theorem 1

For $\eta \in (1,2)$ the unique solution to (3.11) with initial data $\widehat{f}(0)=2\eta$ and $\displaystyle \frac{\textrm{d} \widehat{f}}{\textrm{d} X} (0)=0$ is global (that is, defined for all $X \in {\mathbb R}$), with damped oscillations for $X<0$ and amplified oscillations for $X>0$. More precisely, the solution admits infinitely many local extrema, arranged in a strictly increasing sequence $\{X_k\}_{k \in {\mathbb Z}}$ with $X_0=0$, $\lim \limits _{k \rightarrow \pm \infty } X_k=\pm \infty$, and such that the solution is strictly monotone between the interlacing local positive maxima at $\{X_{2k}\}_{k \in {\mathbb Z}}$ and the local negative minima at $\{X_{2k+1}\}_{k \in {\mathbb Z}}$. Moreover, $\lim \limits _{k \rightarrow -\infty } \widehat{f}(X_k)=0$ and $\lim \limits _{k \rightarrow \infty } \widehat{f}(X_k)=\infty$, with $|\widehat{f}(X_k)|$ strictly increasing as $k \in {\mathbb Z}$ increases.

Writing (3.11) in the equivalent form

**Fig. 2:**
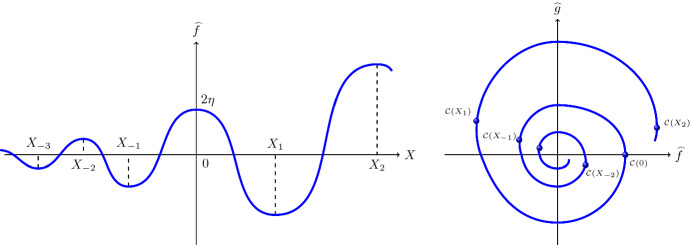
Sketch of the oscillating behaviour of the solution $X \mapsto \widehat{f}(X)$ in Theorem 1. The figure on the left corresponds to the spiralling pattern of the solution curve $X \mapsto {\mathcal C}(X)=(\widehat{f}(X),\,\widehat{g}(X))$ depicted on the right in the phase-space

4.1 $$\begin{aligned} {\left\{ \begin{array}{ll} \displaystyle \frac{\textrm{d} \widehat{f}}{\textrm{d} X} &{}= \widehat{g} -\frac{1}{2}\,\widehat{f}^2 + \eta \,\widehat{f}\,,\\ \displaystyle \frac{\textrm{d} \widehat{g}}{\textrm{d} X} &{}= - \widehat{f}\,, \end{array}\right. } \end{aligned}$$

we see that Theorem 1 ensures that a specific solution of (4.1) spirals in towards the unique equilibrium point at the origin for $X \rightarrow -\infty$, and spirals out from it for $X \rightarrow \infty$; see Fig. 2. Since by uniqueness the solution curves cannot cross, this, in combination with the behaviour of the vector field along the two nullclines (see below), shows that every single non-trivial solution curve of (4.1) traces a similar spiralling pattern.

### Theorem 2

For $\eta \in (1,2)$ all non-trivial solutions to (3.11) feature damped oscillations for $X \rightarrow -\infty$ and amplified oscillations with ultimately unbounded amplitudes for $X \rightarrow \infty$.

### Proof

To establish the solution behaviour claimed in Theorem 1, it is convenient to study (3.11) for $X <0$ by reversing the flow direction. More precisely, setting

4.2 $$\begin{aligned} f(X)=\widehat{f}(-X)\,,\qquad X > 0\,, \end{aligned}$$

we transform (3.11) for $X<0$ into

4.3 $$\begin{aligned} \frac{\textrm{d}^2 f}{\textrm{d} X^2} - (f-\eta )\,\displaystyle \frac{\textrm{d} f}{\textrm{d} X} + f=0\quad \text {for}\quad X > 0\,. \end{aligned}$$

and determine the behaviour of its solutions by investigating the equivalent planar system

4.4 $$\begin{aligned} {\left\{ \begin{array}{ll} \displaystyle \frac{\textrm{d} f}{\textrm{d} X} &{}= g +\frac{1}{2}\,f^2 - \eta f\,,\\ \displaystyle \frac{\textrm{d} g}{\textrm{d} X} &{}= -f\,, \end{array}\right. }\qquad X>0\,. \end{aligned}$$

Since the characteristic equation for the linearisation

4.5 $$\begin{aligned} {\left\{ \begin{array}{ll} \displaystyle \frac{\textrm{d} \widehat{f}}{\textrm{d} X} &{}= \widehat{g} + \eta \, \widehat{f}\,,\\ \displaystyle \frac{\textrm{d} \widehat{g}}{\textrm{d} X} &{}= -\,\widehat{f}\,, \end{array}\right. } \end{aligned}$$

of the system (4.1) near the origin is $\lambda ^2 - \eta \lambda + 1=0$, with eigenvalues $\lambda _{1,2}=\frac{\eta \pm \textrm{i} \sqrt{4-\eta ^2}}{2}$, and that of the linearisation

4.6 $$\begin{aligned} {\left\{ \begin{array}{ll} \displaystyle \frac{\textrm{d} f}{\textrm{d} X} &{}= g - \eta \, f\,,\\ \displaystyle \frac{\textrm{d} g}{\textrm{d} X} &{}= -f\,, \end{array}\right. } \end{aligned}$$

of the system (4.4) near the origin is $\lambda ^2 + \eta \lambda + 1=0$, with eigenvalues $\lambda _{1,2}=\frac{-\eta \pm \textrm{i} \sqrt{4-\eta ^2}}{2}$, the Hartman-Grobman theorem (see [3, 10]) applies. Thus, since $1<\eta <2$, the origin is a repelling focus for the nonlinear system (4.1) and an attractive focus for the nonlinear system (4.4). This confirms, in part, the behaviour of the solution claimed by Theorem 1, but the detailed global pattern specified therein requires a more detailed investigation, based on an interplay of phase-plane analysis and the construction of suitable Lyapunov functions. It turns out that the analysis for (4.4) is rather routine, whereas tackling (4.1) is quite intricate.

The vertical nullcline of (4.4) is the parabola $[g=\eta f -\frac{1}{2}\,f^2]$, along which the vector field points vertically upwards in the left half-plane $[f<0]$ and vertically downwards in the right half-plane $[f>0]$. The horizontal nullcline of (4.4) is the axis $[f=0]$, with the vector field pointing to the right along the positive semi-axis and to the left on the negative semi-axis. Since the origin is the only equilibrium point of (4.4), and solution trajectories are disjoint by uniqueness, this information suffices to infer that the claimed spiralling behaviour of the nontrivial solutions to (3.11) for $X<0$ follows once we establish it for a single solution. With this aim, we now investigate the behaviour of the unique solution curve ${\mathcal C}(X)=(f(X),\,g(X))$ of (4.4) with ${\mathcal C}(0)=(2\eta ,0)$, that is, with

4.7 $$\begin{aligned} f(0)=2\eta \quad \text {and}\quad g(0)=0 \end{aligned}$$

**Fig. 3:**
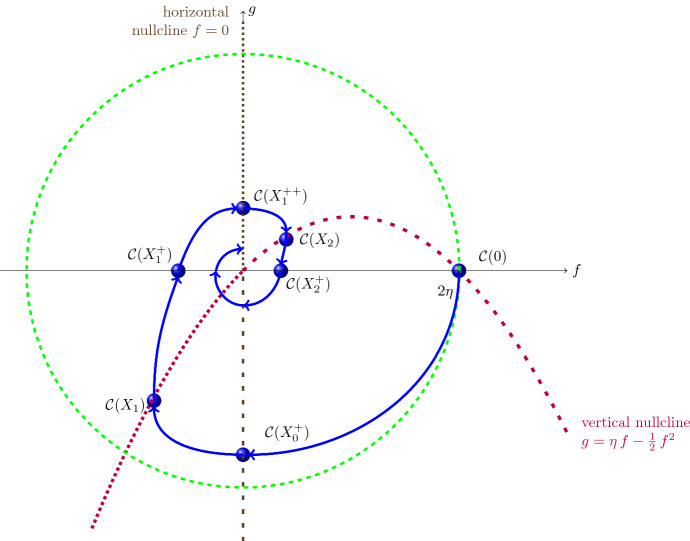
The solution curve $X \mapsto {\mathcal C}(X)$ of the nonlinear system (4.4) through $(0,2\eta )$ spirals around the origin (the single equilibrium point of the system), with continuously diminishing distance from it as *X* grows towards $\infty$. The vector field points vertically upwards along the dotted branch of the vertical isocline in the half-plane $[f<0]$, vertically downward along the dashed branch in the half-plane $[f>0]$, and horizontally along the vertical axis $f=0$ (to the right on the dotted positive semi-axis and to the left on the dashed negative semi-axis). The interior ${\mathcal O}$ of the disk $[f^2 + g^2=4\eta ^2]$ is positively invariant. The connection to Fig. 2 is made by direction reversal, with $X_1$ and $X_2$ corresponding to $X_{-1}$ and $X_{-2}$ in Fig. 2

$\left( \text {so } \displaystyle \frac{\textrm{d} f}{\textrm{d} X}(0)=0\right)$, defined on its maximal existence interval $(X^*_-,\,X^*_+)$, with $X^*_+>0$ and $X^*_-<0$ possibly finite; see Fig. 3. Using the Lyapunov function

4.8 $$\begin{aligned} {\mathcal L}(f,g)= f^2 + g^2 \end{aligned}$$

with

4.9 $$\begin{aligned} \frac{\textrm{d}}{\textrm{d} X} \,{\mathcal L}(f(X),\,g(X)) = f^2(X)\,[ f(X)- 2\eta ]\,,\qquad X \in (0,X^*_+)\,, \end{aligned}$$

from (4.7) we deduce that the interior $${\mathcal O}$$ of the circle $$[f^2 + g^2=4\eta ^2]$$ is positively invariant, with $$X \mapsto {\mathcal L}(f(X),\,g(X))$$ decreasing on $$[0,X^*_+)$$. Indeed, the solution curve $${\mathcal C}$$ enters this region since (3.11) and (4.4) yield

$$\begin{aligned}{} & {} \frac{\textrm{d} f}{\textrm{d} X}(0)=0,\quad \frac{\textrm{d}g}{\textrm{d} X}(0)=-2\eta ,\quad \frac{\textrm{d}^2f}{\textrm{d} X^2}(0)=-2\eta ,\quad \frac{\textrm{d}^2 g}{\textrm{d} X^2}(0)=0, \quad \frac{\textrm{d}^3f}{\textrm{d} X^3}(0)=-2\eta ^2,\\{} & {} \quad \frac{\textrm{d}^3g}{\textrm{d} X^3}(0)=2\eta , \end{aligned}$$

so that $f^2(\varepsilon ) + g^2(\varepsilon ) < 4\eta ^2$ for all $\varepsilon >0$ small enough, due to the Taylor expansion

4.10 $$\begin{aligned} f(\varepsilon )=2\eta - \eta \, \varepsilon ^2 - \tfrac{\eta ^2}{3}\,\varepsilon ^3 +\textrm{O}(\varepsilon ^4) \quad \text {and}\quad g(\varepsilon )=-2\eta \, \varepsilon + \tfrac{\eta }{3}\,\varepsilon ^3 + \textrm{O}(\varepsilon ^4) \,. \end{aligned}$$

Also, once inside the region ${\mathcal O}$, throughout which $f^2 < 4\eta ^2$ (and thus $-2\eta<f<2\eta$), the relation (4.9) ensures that $X \mapsto {\mathcal L}(f(X),\,g(X))$ decreases with growing *X*. In particular, the solution remains bounded and thus blow-up at finite $X>0$ is not possible, so $X^*_+=\infty$.

Since in the region $[f>0,\,g<0] \cap {\mathcal O}$, where by (4.10) the solution curve $X \mapsto {\mathcal C}(X)$ is located for $X>0$ small enough, the functions $X \mapsto f(X)$ and $X \mapsto g(X)$ are strictly decreasing, due to (4.4), with $X \mapsto f^2(X)+g^2(X)$ decreasing by (4.9), we deduce that the solution curve hits first the negative semi-axis $[f=0,\, g<0]$ at some $X_0^+>0$, with

$$\begin{aligned} 0> g(X_0^+) > - 2\eta \quad \text {and}\quad f(X_0^+)=0. \end{aligned}$$

Now (4.4) yields $\frac{\textrm{d}f}{\textrm{d} X}(X_0^+) <0$ and $\frac{\textrm{d}g}{\textrm{d} X}(X_0^+)=0$, so the solution curve will enter the region $[f<0,\,g<0] \cap {\mathcal O}$ for $X>X_0^+$ small enough. It is impossible for the solution curve to remain below the parabola $[g=\eta \,f -\frac{1}{2}\,f^2]$ for all $X>X_0^+$ since then $X \mapsto f(X)$ would be strictly decreasing on $[X_0^+,\infty )$ and

$$\begin{aligned} \frac{\textrm{d}g}{\textrm{d} X}(X) =-f(X) \ge - f(X_0^+ +1),\qquad X \ge X_0^+ +1, \end{aligned}$$

would make *g*(*X*) positive for $X>X_0^+ +1$ large enough. Thus we can find $X_1>X_0^+$ so that the solution curve intersects $[g=\eta f -\frac{1}{2}\,f^2]$ with $f(X_1)<0$ and $g(x_1)<0$, crossing the parabola vertically upwards as $\frac{\textrm{d} f}{\textrm{d} X}(X_1)=0$ and $\frac{\textrm{d}g}{\textrm{d} X}(X_1)=-f(X_1)>0$. The solution curve therefore enters the region $[f<0,\,0>g>\eta f -\frac{1}{2}\,f^2] \cap {\mathcal O}$ for $X>X_1$. Throughout this region, (4.4) yields that $X \mapsto f(X)$ and $X \mapsto g(X)$ are strictly increasing. The solution curve cannot reach the origin at finite *X* (since then, by uniqueness, it would have to be stationary for all values of $X \in {\mathbb R}$) and it also cannot reach the origin as $X \rightarrow \infty$ while confined to this region (since the Hartman-Grobman theorem ensures damped oscillations of *f*(*X*) close enough to the origin). Consequently there exists $X_1^+>X_1$ where the solution hits the negative semi-axis: $g(X_1^+)=0$ and $0>f(X_1^+) > g(X_0^+)$, with the inequality on the right side ensured by the fact that $X \mapsto {\mathcal L}(f(X),g(X))$ is strictly decreasing on $(X_0^+,X_1^+)$. From (4.4) we see that $\frac{\textrm{d}f}{\textrm{d} X}(X_1^+) >0$ and $\frac{\textrm{d}g}{\textrm{d} X}(X_1^+) >0$, so that the solution curve enters the region $[f<0,\,g>0] \cap {\mathcal O}$ for $X>X_1^+$ small enough. In this region, (4.4) ensures that $X \mapsto f(X)$ and $X \mapsto g(X)$ are strictly increasing. Again, the origin cannot be reached in this region at finite *X* or for $X \rightarrow \infty$, so that there is some $X_1^{++}>X_1^+$ where the solution curve hits the positive vertical semi-axis: $f(X_1^{++})=0$ and $0< g(X_1^{++}) <- f(X_1^+)$, with the last inequality a consequence of the monotonicity of $X \mapsto {\mathcal L}(f(X),g(X))$ on $(X_1^+,X_1^{++})$. From (4.4) we now see that $\frac{\textrm{d}f}{\textrm{d} X}(X_1^{++}) > 0$ and $\frac{\textrm{d}g}{\textrm{d} X}(X_1^{++})=0$, so that the solution curve enters the region $[f>0,\,g>0] \cap {\mathcal O}$ for $X>X_1^{++}$ small enough. Throughout this region the solution curve must intersect the vertical nullcline at finite *X* since otherwise (4.4) would yield that $X \mapsto f(X)$ is strictly increasing and $X \mapsto g(X)$ is strictly decreasing on $(X_1^{++},\infty )$, but the origin is the only possible accumulation point in the closure of ${\mathcal O}$. Let $X_2>X_1^{++}$ correspond to the intersection of the solution curve with the vertical isocline. Then $\frac{\textrm{d}f}{\textrm{d} X}(X_2)=0$ and $\frac{\textrm{d}g}{\textrm{d} X}(X_2) <0$, so that the solution enters the region $[f>0,\,0< g < \eta f -\frac{1}{2}\,f^2]$ for $X>X_2$ small enough. In this region, (4.4) ensures that $X \mapsto f(X)$ and $X \mapsto g(X)$ are strictly decreasing, and since, as above, the solution curve cannot reach the origin in this region (at finite *X* or for $X \rightarrow \infty$), there is some $X_2^+ >X_2$ where the solution curve hits the positive horizontal semi-axis: $g(X_2^+)=0$ and $0< f(X_2^+)< g(X_1^{++})$. From (4.4) we now get $\frac{\textrm{d}f}{\textrm{d} X}(X_2^+) <0$ and $\frac{\textrm{d}g}{\textrm{d} X}(X_2^+) <0$, so the solution crosses the positive horizontal semi-axis, entering the region $[f>0,\,g<0] \cap {\mathcal O}$ for $X>X_2^+$ small enough. We now repeat the arguments step-by-step, showing that the solution spirals around the origin, forever getting closer to it as *X* increases. Thus we have proved the claim regarding the behaviour for $X<0$ of the nontrivial solution to (3.11) with initial data

4.11 $$\begin{aligned} \widehat{f}(0)=2\eta \quad \text {and}\quad \frac{\textrm{d} \widehat{f}}{\textrm{d} X}(0)=0\,, \end{aligned}$$

corresponding to the initial data (4.7) for (4.4).

Let us now investigate the behaviour on the maximal existence interval $$[0,-X_-^*)$$ of the solution $$(\widehat{f}(X),\,\widehat{g}(X))$$ to the planar system (4.1) with initial data

4.12 $$\begin{aligned} \widehat{f}(0)=2\eta \quad \text {and}\quad \widehat{g}(0)=0\,, \end{aligned}$$

corresponding to the initial data

4.13 $$\begin{aligned} \widehat{f}(0)=2\eta \quad \text {and}\quad \frac{\textrm{d}\widehat{f}}{\textrm{d} X}(0)=0 \end{aligned}$$

for (3.11). Rather than working with the system (4.1), it is convenient to transform it by means of

4.14 $$\begin{aligned} {\left\{ \begin{array}{ll} f(X) = -\widehat{f}(X)\,,\\ g(X)= -\widehat{g}(X)\,, \end{array}\right. } \qquad X \ge 0\,, \end{aligned}$$

into the equivalent system

4.15 $$\begin{aligned} {\left\{ \begin{array}{ll} \displaystyle \frac{\textrm{d} f}{\textrm{d} X} &{}= g +\frac{1}{2}\,f^2 + \eta f\,,\\ \displaystyle \frac{\textrm{d} g}{\textrm{d} X} &{}= -f\,, \end{array}\right. }\qquad X>0\,. \end{aligned}$$

with initial data

4.16 $$\begin{aligned} f(0)=-2\eta \quad \text {and}\quad g(0)=0\,. \end{aligned}$$

Analogous to (4.8), a useful Lyapunov function for (4.15) is

4.17 $$\begin{aligned} {\mathcal L}_1(f,g)= f^2 + g^2 \end{aligned}$$

with

4.18 $$\begin{aligned} \frac{\textrm{d}}{\textrm{d} X} \,{\mathcal L}_1(f(X),g(X)) = f^2(X)\,[2\eta + f(X)]\,,\qquad X \in (0,-X_-^*)\,. \end{aligned}$$

Note that (4.15) and (4.16) yield

$$\begin{aligned}{} & {} \frac{\textrm{d} f}{\textrm{d}X}(0)=0,\quad \frac{\textrm{d} g}{\textrm{d}X}(0)=2\eta ,\quad \frac{\textrm{d}^2 f}{\textrm{d}X^2}(0)=2\eta ,\quad \frac{\textrm{d}^2 g}{\textrm{d}X^2}(0)=0,\quad \frac{\textrm{d}^3 f}{\textrm{d}X^3}(0)=-2\eta ^2 ,\\{} & {} \quad \quad \frac{\textrm{d}^3 g}{\textrm{d}X^3}(0)=-2\eta , \end{aligned}$$

so that $f^2(\varepsilon ) + g^2(\varepsilon ) > 4\eta ^2$ for $\varepsilon >0$ small enough, since

4.19 $$\begin{aligned} f(\varepsilon )=-2\eta + \eta \, \varepsilon ^2 - \tfrac{\eta ^2}{3}\,\varepsilon ^3 + \textrm{O}(\varepsilon ^4) \quad \text {and}\quad g(\varepsilon )=2\eta \, \varepsilon - \tfrac{\eta }{3}\,\varepsilon ^3 + \textrm{O}(\varepsilon ^3) \,. \end{aligned}$$

**Fig. 4:**
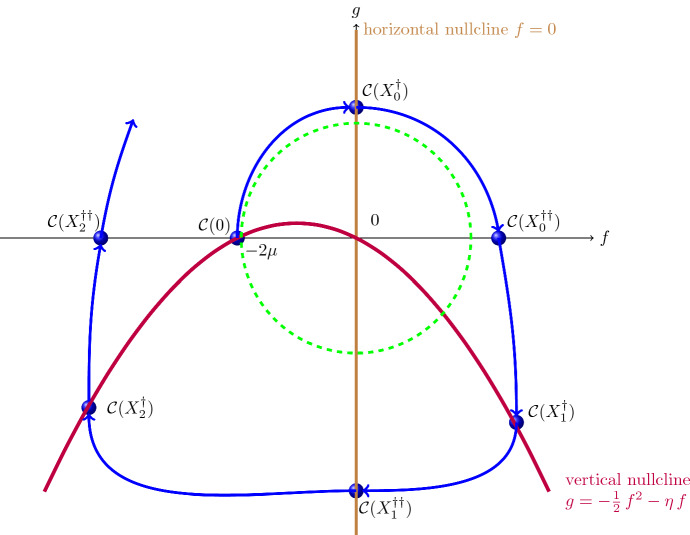
Behaviour of the solution to (4.15) with initial data (4.16). The connection to Fig. 2 is made by means of the transformation (4.14) and noticing that $X_1^\dagger$ and $X_2^\dagger$ correspond to $X_{1}$ and $X_{2}$ in Fig. 2, respectively

These considerations, in combination with (4.18), show that the solution curve starting at the point $(-2\eta ,0)$ on the boundary of the disk $[f^2+g^2 = 4\eta ^2]$ is confined to the exterior of this disk for all $X \in (0,-X_-^*)$. From (4.19) we see that the solution curve enters the quadrant $[f<0,\,g>0]$ for $X>0$ small enough, and throughout this quadrant $X \mapsto f(X)$ and $X \mapsto g(X)$ are both strictly increasing, with the second statement a consequence of the fact that, in this region, any point (*f*, *g*) with $f^2+g^2>4\eta ^2$ lies above the graph of the parabola $g=-\frac{1}{2}\,f^2 - \eta \,f$ (see Fig. 4). We claim that there is some $X_0^\dagger \in (0,-X_-^*)$ where the solution curve hits the positive vertical semi-axis:

$$\begin{aligned} f(X_0^\dagger )=0 \quad \text {and}\quad g(X_0^\dagger ) > 2\eta . \end{aligned}$$

Indeed, otherwise, by choosing $\varepsilon >0$ small enough, we could ensure that

$$\begin{aligned} -2\eta< f(\varepsilon ) \le f(X)<0 \quad \text {and}\quad 0 < g(\varepsilon ) \le g(X) \quad \text {for all}\quad X \in (\varepsilon ,-X_-^*). \end{aligned}$$

But then integration of (4.18) on $[\varepsilon ,X]$ for $X \in (\varepsilon ,-X_-^*)$ prevents blow-up at finite *X*, so $-X_-^*=\infty$, which is incompatible with the solution curve remaining in this quadrant since then

$$\begin{aligned} g(X) + \tfrac{1}{2}\,f^2(X) + \eta \,f(X)> g(X) \ge g(\varepsilon ) >0,\qquad X \ge \varepsilon , \end{aligned}$$

so that integrating the first equation in (4.15) on $[\varepsilon ,X]$ would ensure that $f(X^*)=0$ for some $X^*>\varepsilon$.

At $X=X_0^\dagger$ we get from (4.15) that $\frac{\textrm{d} f}{\textrm{d}X}(X_0^\dagger )>0$ and $\frac{\textrm{d} g}{\textrm{d}X}(X_0^\dagger )=0$, so that the solution curve enters the quarter-plane $[f>0,\,g>0]$ for $X>X_0^\dagger$, with its distance from the origin increasing with growing *X*, due to (4.18). In this quarter-plane $X \mapsto f(X)$ is increasing while $X \mapsto g(X)$ decreases. We claim that there is some $X_0^{\dagger \dagger }>X_0^\dagger$ where the solution hits the positive horizontal semi-axis:

4.20 $$\begin{aligned} g(X_0^{\dagger \dagger })=0 \quad \text {and}\quad f(X_0^{\dagger \dagger })> g(X_0^{\dagger })>2\eta \,, \end{aligned}$$

when we also take (4.18) into account. Indeed, to see that blow-up at finite *X* is not possible in this quarter-plane, we consider the Lyapunov function

4.21 $$\begin{aligned} {\mathcal L}_2(f,g)= f\,\textrm{e}^{g/2} \end{aligned}$$

with

4.22 $$\begin{aligned} \frac{\textrm{d}}{\textrm{d} X} \,{\mathcal L}_2(f(X),g(X)) = \eta \,{\mathcal L}_2(f(X),g(X)) + g(X)\,\textrm{e}^{g(X)/2}\,,\qquad X \in (0,-X_-^*)\,. \end{aligned}$$

If the solution curve were to remain in the quarter-plane $[f>0,\,g>0]$ for all $X>X_0^\dagger$, then $0 < g(X) \le g(X_0^\dagger )$ for all $X>X_0^\dagger$ and (4.22) would give

$$\begin{aligned} \frac{\textrm{d}}{\textrm{d} X} \,{\mathcal L}_2(f(X),g(X)) \le \eta \,{\mathcal L}_2(f(X),g(X)) + g(X_0^\dagger )\,\textrm{e}^{g(X_0^\dagger )/2},\qquad X \in (X_0^\dagger ,-X_-^*), \end{aligned}$$

so that the Gronwall inequality would yield the boundedness of ${\mathcal L}_2(f(X),g(X))$ on the finite interval $[X_0^\dagger ,-X_-^*)$. Knowing already that *g*(*X*) is bounded, *f*(*X*) would also be bounded, thus preventing blow-up. We can also rule out the possibility that the solution is defined for all $X> X_0^\dagger$ and the solution curve remains in the quarter-plane $[f>0,\,g>0]$ for all $X>X_0^\dagger$, since the monotonicity of $X \mapsto f(X)$ ensures $f(X) \ge f(X_0^\dagger +1)>0$ for all $X>X_0^\dagger +1$, so that by integrating the second equation in (4.15) we would obtain

$$\begin{aligned} g(X) \le g(X_0^\dagger +1) - [X-X_0^\dagger -1]\, f(X_0^\dagger +1),\qquad X \ge X_0^\dagger +1, \end{aligned}$$

and this means that *g*(*X*) must vanish at finite *X*. Consequently there is some $X_0^{\dagger \dagger } \in (X_0^{\dagger },-X_-^*)$ where the relations specified in (4.20) hold. From (4.15) we now see that $\frac{\textrm{d}f}{\textrm{d}X}(X_0^{\dagger \dagger })>0$ and $\frac{\textrm{d}g}{\textrm{d}X}(X_0^{\dagger \dagger }) < 0$, so that the solution curve enters the quarter-plane $[f>0,\,g<0]$ for $X>X_0^{\dagger \dagger }$, getting, by (4.18), further away from the origin with growing *X*. We now show that the solution cannot blow-up at finite *X* while remaining in this quarter-plane. Indeed, since the function $g \mapsto g\,\textrm{e}^{g}$ is bounded on $(-\infty ,0]$, Gronwall’s inequality ensures, by means of relation (4.22), that ${\mathcal L}_2(f(X),g(X))$ remains bounded in the presumedly finite interval $[X_0^{\dagger \dagger },-X_-^*)$. Thus

$$\begin{aligned} f(X)\,\textrm{e}^{g(X)/2}= - \frac{\textrm{d}g}{\textrm{d}X}(X)\,\,\textrm{e}^{g(X)/2} \end{aligned}$$

remains bounded on $(X_0^{\dagger \dagger },-X_-^*)$, which integrated yields the boundedness of $X \mapsto g(X)$ on a finite interval $[X_0^{\dagger \dagger },-X_-^*)$. Because ${\mathcal L}_2(f(X),g(X))=f(X)\,\textrm{e}^{g(X)/2}$ is also bounded, $X \mapsto f(X)$ has to be bounded on a finite interval $[X_0^{\dagger \dagger },-X_-^*)$, preventing the assumed blow-up at finite *X*.

We now claim that the solution curve must intersect the vertical nullcline $[g=-\frac{1}{2}\,f^2 - \mu f]$ at finite *X* in the quarter-plane $[f>0,\,g<0]$; otherwise we would have

4.23 $$\begin{aligned} {\left\{ \begin{array}{ll} g(X) + \frac{1}{2}\,f^2(X) + \eta \, f(X)>0\,,\\ f(X)>0> g(X)\,, \end{array}\right. }\qquad X \in (X_0^{\dagger \dagger },-X_-^*)\,. \end{aligned}$$

We now introduce the Lyapunov function

4.24 $$\begin{aligned} {\mathcal L}_3(f,g)= g + \eta \, f \end{aligned}$$

with

4.25 $$\begin{aligned}{} & {} \frac{\textrm{d}}{\textrm{d} X} \,{\mathcal L}_3(f(X),g(X)) = \eta \,{\mathcal L}_3(f(X),g(X)) + f(X)\,\Big ( \tfrac{\eta }{2}\,f(X) -1\Big ) > \eta \,{\mathcal L}_3(f(X),g(X)),\nonumber \\{} & {} \qquad X \in (X_0^{\dagger \dagger },-X_-^*), \end{aligned}$$

if (4.23) holds, since then the first equation in (4.15) would ensure, in view of the assumption (4.23), that $X \mapsto f(X)$ is increasing for $X>X_0^{\dagger \dagger }$, and $f(X_0^{\dagger \dagger })>2\eta$ forces $\frac{\eta }{2}\,f(X_0^{\dagger \dagger }) -1>0$ because $\eta >1$. Now, taking advantage of the fact that ${\mathcal L}_3(f(X_0^{\dagger \dagger }),g(X_0^{\dagger \dagger })) >0$, from the differential inequality (4.25) we would get

$$\begin{aligned} {\mathcal L}_3(f(X),g(X)) =g(X) + \eta \,f(X) >0,\qquad X \ge X_0^{\dagger \dagger }, \end{aligned}$$

and the first equation in (4.15) would lead us to

$$\begin{aligned} \frac{\textrm{d}f}{\textrm{d}X}(X)> \tfrac{1}{2} \,f^2(X),\qquad X > X_0^{\dagger \dagger }, \end{aligned}$$

with $f(X) \ge f(X_0^{\dagger \dagger }) >2\eta$. Dividing both sides of the above differential inequality by $f^2(X)$ and integrating, we would obtain the inequality

$$\begin{aligned} \frac{1}{f(X_0^{\dagger \dagger })}> \frac{1}{f(X_0^{\dagger \dagger })} - \frac{1}{f(X)}> \tfrac{1}{2} \,(X-X_0^{\dagger \dagger }),\qquad X > X_0^{\dagger \dagger }, \end{aligned}$$

which clearly fails for $X>X_0^{\dagger \dagger }$ large enough. Consequently there is some $X_1^\dagger \in (X_0^{\dagger \dagger },-X_-^*)$ with

4.26 $$\begin{aligned} f(X_1^\dagger )>0>g(X_1^\dagger ) =- \tfrac{1}{2}\,f^2(X_1^\dagger ) - \eta f(X_1^\dagger )\,. \end{aligned}$$

From (4.15) we see that in the quarter-plane $[f>0,\, g<0]$ the solution curve crosses the vertical isocline vertically downwards, so that it moves, for $X>X_1^\dagger$, into the region ${\mathcal R}= [f>0,\, g < - \tfrac{1}{2}\,f^2 - \eta \, f]$, in which both $X \mapsto f(X)$ and $X \mapsto g(X)$ are strictly decreasing. For $\varepsilon >0$ small enough we then have

$$\begin{aligned} E= - [g(X_1^\dagger + \varepsilon ) + \tfrac{1}{2} \,f^2(X_1^\dagger +\varepsilon ) + \eta \, f(X_1^\dagger +\varepsilon )] > 0, \end{aligned}$$

with the monotonicity properties ensuring that $g(X) + \tfrac{1}{2} \,f^2(X) + \eta \, f(X) \le -E$ for all $X>X_1^\dagger + \varepsilon$ for which the solution lies in the region ${\mathcal R}$. From (4.15) we now infer $\frac{\textrm{d}f}{\textrm{d} X}(X) \le -E$ and $0> \frac{\textrm{d}g}{\textrm{d} X}(X) > - f(X_1^\dagger + \varepsilon )$ for all $X>X_1^\dagger + \varepsilon$ for which the solution lies in the region ${\mathcal R}$, so that blow-up at finite *X* is prevented and the solution curve must intersect the negative vertical semi-axis at finite *X*: there exists some $X_1^{\dagger \dagger } \in (X_1^\dagger ,-X_-^*)$ with

$$\begin{aligned} f(X_1^{\dagger \dagger })=0 \quad \text {and}\quad g(X_1^{\dagger \dagger })< -f(X_0^{\dagger \dagger }) <-2\eta . \end{aligned}$$

Since (4.15) yields $\frac{\textrm{d}f}{\textrm{d} X}(X_1^{\dagger \dagger })<0$ and $\frac{\textrm{d}g}{\textrm{d} X}(X_1^{\dagger \dagger })=0$, the solution curve enters the region ${\mathcal U}=[f<0,\,g < -\frac{1}{2}\,f^2 - \eta \,f]$, throughout which, due to (4.18), it satisfies $f^2(X)+g^2(X) \ge g^2(X_1^{\dagger \dagger }) > 4\eta ^2$, with $X \mapsto f(X)$ strictly decreasing and $X \mapsto g(X)$ strictly increasing. Consequently the solution curve must hit the vertical nullcline at some $X_2^\dagger > X_1^{\dagger \dagger }$, with

$$\begin{aligned} f(X_2^\dagger )<0 \quad \text {and}\quad g(X_2^\dagger )=- \tfrac{1}{2} \,f^2(X_2^\dagger ) - \eta \, f(X_2^\dagger ) <0, \end{aligned}$$

while (4.15) ensures $\frac{\textrm{d}f}{\textrm{d} X}(X_2^\dagger )=0$ and $\frac{\textrm{d}g}{\textrm{d} X}(X_2^\dagger )>0$, so that the solution curve crosses the nullcline vertically upwards at $X=X_2^\dagger$. Since the vector field points vertically upwards all along the vertical nullcline in the quarter-plane $[f<0,\,g<0]$, we see that the solution must lie above the curve $[g+ \tfrac{1}{2} \,f^2 + \eta \, f=0]$ for $X>X_2^\dagger$, as long as $f(X)<0$ and $g(X)<0$. Therefore, the solution curve must hit the negative horizontal semi-axis at some $X_2^{\dagger \dagger }>X_2^\dagger$, with

$$\begin{aligned} g(X_2^{\dagger \dagger })=0 \quad \text {and}\quad f(X_2^{\dagger \dagger })< g(X_1^{\dagger \dagger }) < -2\eta . \end{aligned}$$

From (4.15) we see that $\frac{\textrm{d}f}{\textrm{d} X}(X_2^{\dagger \dagger })>0$ and $\frac{\textrm{d}g}{\textrm{d} X}(X_2^{\dagger \dagger })>0$, so that the solution curve enters the quarter-plane $[f<0,\,g>0]$, where it cannot cross the solution path $( f(X),\,g(X))_{0< X < X_0^\dagger }$, so that in this region we have $\frac{\textrm{d}f}{\textrm{d} X}(X)>0$ and $\frac{\textrm{d}g}{\textrm{d} X}(X) >0$ along the solution curve. We can now repeat the arguments used before to infer that the solution curve hits the positive vertical semi-axis, then enters the quarter-plane $[f>0,\,g>0]$ and goes around the origin, exactly as for the case $X \in [0,X_2^{\dagger \dagger }]$, but now further away from the origin, due to (4.18). We conclude that the solution curve exists for all $X>0$, and spirals around (and away) from the origin, as claimed. $\square$

Thus we have a decaying oscillation in $X>0$ and, with the same initial conditions for Eq. (3.11), a growing oscillation in $X<0$; this result must now be embedded within a description that corresponds to the observed properties of the morning glory. It is immediately clear that we cannot permit the growing solution, but we could treat the decaying oscillation as starting from any trough (at a finite point) and evolving to the left; the solution to the right must take a different form. How this is accomplished we describe in the next section.

## The undular bore

The first stage in the construction of a solution is quite straightforward: we make choices for *a*(*z*) and *b*(*z*) in (3.1), using (3.2)–(3.4). The simplest way to proceed is to set

5.1 $$\begin{aligned} b(z)=b_0 + b_1\,\textrm{e}^{-Bz}\,, \end{aligned}$$

where $b_0$, $b_1$ and *B* are constants (and we require $B>0$ since at great heights – near the tropopause – this type of atmospheric flow is barely noticeable); other choices are possible, but this one is computationally advantageous. Equation (3.4) then gives

5.2 $$\begin{aligned} M(z)=rb_0B\,, \end{aligned}$$

which specifies the horizontal kinematic eddy viscosity. Equation (3.3) now produces an expression for the vertical dynamic eddy viscosity

5.3 $$\begin{aligned} m(z)=A \,\textrm{e}^{Bz} + \frac{(qb_0 -B)b_1}{B^2}\,, \end{aligned}$$

where *A* is an arbitrary real constant. The various constants here can be chosen to model the viscosity, at least over the vertical extent of the undular bore; in particular, the choice $A=0$ gives *m* constant. Finally, we use equation (3.2) to obtain an expression for *a*(*z*), to produce the associated heating source in the form

5.4 $$\begin{aligned} k(z)=- k_0 \Big \{b_0b_1\,\textrm{e}^{-Bz} + \beta \,\frac{\textrm{d}a}{\textrm{d}z} +\frac{\textrm{d}}{\textrm{d}z}\Big ( m\, \frac{\textrm{d}a}{\textrm{d}z} \Big )\Big \}\,, \end{aligned}$$

due to (3.5). We now turn to the all-important issue of finding suitable functions *F* for stream functions of type (3.1).

The considerations in Sects. 3 and 4 make clear that the construction of a suitable function *F* will require some care. Indeed, a description of the whole flow field is a significant challenge, but showing that an undular-bore profile exists is attainable. To do this we concentrate our efforts on the streamline that defines the front of the wave and its associated oscillations. Let the streamline that is this profile be given by $\psi (\xi ,y)=0$, then the equation for its shape is

5.5 $$\begin{aligned} a(z) + F_0 b(z)\, f(X)=0\,, \end{aligned}$$

written in terms of *z* and $X=\xi /\xi _0=(x-ct)/\xi _0$, where $F_0 \ne 0$ and $\xi _0>0$ are constants (introduced in Sect. 3). The function which controls the shape of the wave, *f*(*X*), satisfies

5.6 $$\begin{aligned} \frac{\textrm{d}^2 f}{\textrm{d} X^2} + f\,\,\frac{\textrm{d} f}{\textrm{d} X} + f+ \nu \,\frac{\textrm{d} f}{\textrm{d} X}= \kappa \,; \end{aligned}$$

see Eq. (3.7), where we have set $\text {sgn}(rq)=1$. We seek a solution of Eq. (5.6) for which $f \rightarrow 0$ as $X \rightarrow \infty$ (ahead of the wave front) and $f \rightarrow f_0>0$ as $X \rightarrow - \infty$ (behind the front, where oscillations die out). Let the corresponding solutions of (5.5) be $z=z_\pm$, so that

5.7 $$\begin{aligned} a(z_+)=0\,,\qquad a(z_-) + F_0 f_0 \,b(z_-)=0\,. \end{aligned}$$

Further, consider a point $(X,z)=(X_0,z_0)$ on the leading face of the bore, and then

**Fig. 5:**
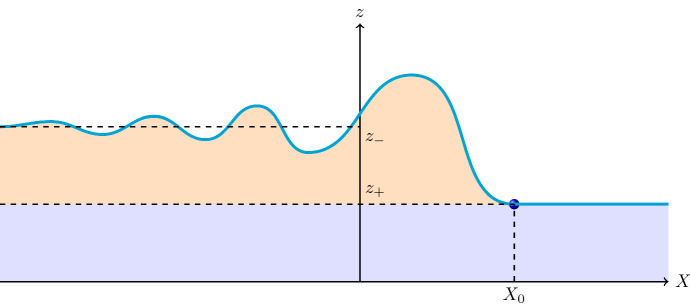
Sketch of an undular bore, generated when a thermal inversion occurs – with the layer of colder air (depicted in blue) overlain by a layer of warmer air (depicted in orange)

5.8 $$\begin{aligned} a(z_0) + F_0 f_0 \,b(z_0)=0\,; \end{aligned}$$

see Fig. 5. The analysis performed in Sect. 4 shows that a solution to (5.6) which oscillates about $f=f_0>0$ for $X \rightarrow -\infty$, that is, with $\kappa =f_0$, cannot satisfy decay conditions ahead of the front (as $X \rightarrow \infty$); conversely, a solution which does decay ahead (so $\kappa =0$) cannot exhibit decaying oscillations about $f=f_0>0$ as $X \rightarrow -\infty$. We must, perforce, construct a solution which satisfies different versions of (5.6) in different regions.

In the light of the situation described above, we consider two regions: $X>X_0$ and $X<X_0$; we expect some form of discontinuity across $X=X_0$ but, as we shall see, this can be regarded as consistent with the general framework that corresponds to the generation of morning-glory waves. Thus we examine, for $f_0>0$, the equation

5.9 $$\begin{aligned} \frac{\textrm{d}^2 f}{\textrm{d} X^2} + f\,\,\frac{\textrm{d} f}{\textrm{d} X} + f+ \nu \,\frac{\textrm{d} f}{\textrm{d} X}={\left\{ \begin{array}{ll} f_0 \,,\quad X<X_0\,,\\ 0\,,\quad X>X_0\,, \end{array}\right. }\,. \end{aligned}$$

The results in Sect. 4 show that in the region $X<X_0$ there are solutions (which depend on the parameter $\nu$, but with $-2<f_0+\nu <0$) which satisfy

$$\begin{aligned} f(X_0)=\frac{\textrm{d} f}{\textrm{d} X}(X_0)=0 \quad \text {and}\quad \frac{\textrm{d}^2 f}{\textrm{d} X^2}(X_0)=f_0 \end{aligned}$$

at some $X_0 \in {\mathbb R}$, and which oscillate around $f=f_0$ and decay as $X \rightarrow -\infty$. Correspondingly, there is the solution $f \equiv 0$ in $X>X_0$; it is convenient hereafter to set $X_0=0$ (see Fig. 5). Thus the profile described by (5.9), with the conditions just outlined, is a continuously differentiable function on ${\mathbb R}$. The jump discontinuity of $\frac{\textrm{d}^2 f}{\textrm{d} X^2}$ across $X=X_0=0$, which relates directly to the heat-source term, is consistent with what is observed as the morning glory is generated. The front of the wave is at the head of a warm breeze that moves into a colder region of air, riding over a cold breeze: there is a difference in temperature and heating across the front. In our solution, this arises by virtue of the discontinuity in $\frac{\textrm{d}^2 f}{\textrm{d} X^2}$ because this is given by the jump in the forcing constant (from 0 to $f_0$). In terms of the associated stream function (3.1), this jump discontinuity of $\frac{\textrm{d}^2 f}{\textrm{d} X^2}$ across $X=X_0=0$ corresponds to a Dirac-delta singularity of the vorticity, concentrated on a vortex patch.

Now that we have a solution for *f*(*X*) which represents an undular bore, and so recovers the essential structure of the morning glory, we can examine the nature of the velocity field associated with this wave. Although many different avenues and choices could be explored, we consider the simple case described by (5.1) with associated constant values of $M>0$ and $m>0$ in (5.2)-(5.3), while the velocity components are given by (2.7). We impose $w_0=0$ on $z=0$ (i.e., no flow through the surface of the Earth, so $z=0$ is a streamline): $b(0)=0$ and so $b_1=b_0$ which gives

5.10 $$\begin{aligned} b(z)=b_0( 1 - \textrm{e}^{-Bz})\,. \end{aligned}$$

Thus

$$\begin{aligned} w_0(z,X)=-\frac{F_0 b_0( 1 - \textrm{e}^{-Bz})}{\xi _0\, d(z)}\,\frac{\textrm{d} f}{\textrm{d} X} , \end{aligned}$$

and we see that $w_0=0$ whenever $\frac{\textrm{d} f}{\textrm{d} X}=0$, which occurs in $X>0$ and for specific values of $X<0$; otherwise, $w_0$ oscillates as $\frac{\textrm{d} f}{\textrm{d} X}$ oscillates. The horizontal velocity component along $z=0$ can also be found:

5.11 $$\begin{aligned} V_0(0,X)=\frac{C}{C \cos ^2\alpha +\sin ^2\alpha }\,\Big (\frac{\textrm{d} a}{\textrm{d} z}(0) + F_0\, f(X)\,\frac{\textrm{d} b}{\textrm{d} z}(0) \Big )\,, \end{aligned}$$

where

$$\begin{aligned} \frac{\textrm{d} a}{\textrm{d} z}(0)=c + pb_0B,\qquad \frac{\textrm{d} b}{\textrm{d} z}(0) =b_0B, \end{aligned}$$

due to (5.10) and (3.2). A breeze exists along $z=0$ because our choices do not admit a no-slip condition; let it be given by

$$\begin{aligned} V_0(0,X)={\left\{ \begin{array}{ll} V_+ \quad \text {for}\quad X>0,\\ V_- \quad \text {for}\quad X \rightarrow - \infty . \end{array}\right. } \end{aligned}$$

Then (5.11) yields

$$\begin{aligned} V_+=\frac{C(c + pb_0B)}{C \cos ^2\alpha +\sin ^2\alpha },\qquad V_-=\frac{C\big (c + pb_0B\big [1+ \frac{f_0}{\nu }\big ]\big )}{C \cos ^2\alpha +\sin ^2\alpha }, \end{aligned}$$

where, from our earlier considerations, $-2< f_0+\nu <0$. Thus

$$\begin{aligned} V_+ - V_-=\frac{M}{\xi _0}\,\frac{f_0}{\cos ^2\alpha +\frac{\sin ^2\alpha }{C}}, \end{aligned}$$

so that $V_+>V_-$ and

5.12 $$\begin{aligned} c= \frac{(\nu +f_0)V_+ - \nu V_-}{f_0}\, \Big ( \cos ^2\alpha + \frac{\sin ^2\alpha }{C}\Big )\,. \end{aligned}$$

**Fig. 6:**
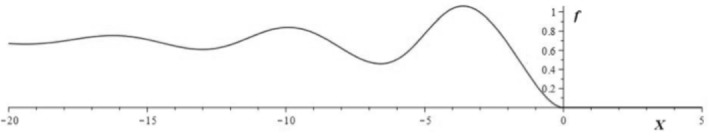
Numerical solution (using Maple) of Eq. (5.9) with $f_0=0.7$, $\nu =-1$ and $X_0=0$. The wave profile propagates to the right with speed $c=\frac{10\,V_- -7\,V_+}{7}\,\big ( \cos ^2\alpha + \frac{\sin ^2\alpha }{C}\big )$, where $V_\pm$ are the breeze speeds

The colliding breezes are defined by the initial state of the atmosphere, before the formation of the morning glory; thus we may presume that we are given $V_+$ and $V_-$. The constants *C*, $\alpha$ and $f_0$ are known from the prescribed state of the atmosphere, and so (5.12) determines the speed of the wave, in terms of the parameter $\nu \in (-2-f_0,-f_0)$; an example of an undular-bore solution of Eq. (5.9) is shown in Fig. 6. The importance of the free parameter $\nu$ is revealed by interpreting (5.12) as a relation between the wave speed and the oscillation amplitude of the bore: knowing *c*, we obtain $\nu$ and the unique solution of the ordinary differential Eq. (5.9) in $X<0$, with initial data $f(0)=\frac{\textrm{d}f}{\textrm{d}X}(0)=0$, which determines the oscillations, the size $f_0$ of the jump being prescribed by the height of the warmer-breeze layer on top of the colder layer which is at ground level.

## Discussion

A consistent theoretical model for atmospheric undular bores was only recently developed (see [8]), while earlier studies rely on rather *ad hoc* simplifying assumptions, reasoning by analogy with shallow-water theory – an approach that fails to address the inherent complexity of the underlying thermodynamical processes. Another shortcoming of earlier attempts to understand these waves is the use of piecemeal models to describe, separately, the core features of the flows (such as jumps, oscillations and the existence of background breezes). So, although these methods have led to the identification of the main ingredients that make-up the morning glory, no complete and relevant solution has been found that combines all the essential elements. In the current work, we remedy this situation and show that a suitable solution does exist. The travelling-wave *Ansatz* leads us to a nonlinear second-order ordinary differential equation – of Liénard type – that looks deceptively simple but has no explicit non-trivial solutions. By means of a quite intricate global analysis in phase-space, which relies on three functionally independent Lyapunov functions, we show that all nontrivial solutions are oscillatory, bounded (and approaching a constant state) in one direction, but unbounded in the other. This confirms the main structure of a damped oscillation at an upper level – an essential ingredient for an atmospheric undular bore – but the streamline which represents the front of the wave should also feature non-oscillatory decay ahead. Consequently different versions of the Liénard equation are required ahead and at-and-behind the front. The discussion of the global dynamics has proved that the oscillating solution exists behind and, emanating from any chosen trough, we may have a constant solution ahead. However, although the resulting solution is continuously differentiable, it possesses a jump in the second derivative at the junction of the two solutions. In the context of the morning glory such a discontinuity is consistent with the underlying physics: the jump in the second derivative comes about by virtue of a jump in the heat-source term. This describes precisely what is observed as a morning-glory wave is generated, that is, a layer of warm air over-rides a layer of colder air and so, at the front, there is a jump in temperature (and in the associated heating). This work, we suggest, therefore lays the foundations for more intensive mathematical investigations as well as careful applications to, and interpretation of, observational data.

## Data Availability

All data for this paper are properly cited and referred to in the reference list.

## References

[1] Berry MV (2018). Minimal analytical model for undular tidal bore profile; quantum and Hawking effect analogies. New J. Phys..

[2] Boyd JP (1990). Weakly nonlocal solitary waves and beyond-all-orders asymptotics. Generalized Solitons and Hyperasymptotic Perturbation Theory.

[3] Bressan A (2007). Tutorial on the center manifold theorem. Hyperbolic Systems of Balance. Laws Lecture Notes in Math.

[4] Clarke RH, Smith RK, Reid DG (1981). The Morning Glory of the Gulf of Carpentaria: an atmospheric undular bore. Mon. Weather Rev..

[5] Constantin A (2011). Nonlinear Water Waves with Applications to Wave-Current Interactions and Tsunamis.

[6] Constantin A, Johnson RS (2021). On the modelling of large-scale atmospheric flow. J. Differ. Equ..

[7] Constantin A, Johnson RS (2021). On the propagation of waves in the atmosphere. Proc. A.

[8] Constantin A, Johnson RS (2022). On the propagation of nonlinear waves in the atmosphere. Proc. A.

[9] Da Silva AFT, Peregrine DH, Edge BL (1990). Nonsteady computations of undular and breaking bores. Proceedings of 22nd Conference on Coastal Engineering.

[10] Dumortier F, Llibre L, Artés JC (2006). Qualitative Theory of Planar Differential Systems.

[11] Haghi KR, Geerts B, Chipilski HG, Johnson A, Degelia S, Imy D, Parsons DB, Adams-Selin RD, Turner DD, Wang X (2019). Boreing into nocturnal convection. Bull. Am. Meteorol. Soc..

[12] Haghi KR, Durran DR (2021). On the dynamics of atmospheric bores. J. Atmos. Sci..

[13] Holton JR, Hakim GJ (2013). An Introduction to Dynamic Meteorology.

[14] Jiang Q (2014). Applicability of reduced-gravity shallow-water theory to atmospheric flow over topography. J. Atmos. Sci..

[15] Johnson RS (1997). A modern introduction to the mathematical theory of water waves.

[16] Johnson RS (1972). Shallow water waves on a viscous fluid the undular bore. Phys. Fluids.

[17] Loveless DM, Wagner TJ, Turner DD, Ackerman SA, Feltz WF (2019). A composite perspective on bore passages during the PECAN campaign. Mon. Weather Rev..

[18] Noonan JA, Smyth NF (1985). Linear and weakly nonlinear internal wave theories applied to “morning glory" waves. Geophys. Astrophys. Fluid Dyn..

[19] Osborne SR, Lapworth A (2017). Initiation and propagation of an atmospheric bore in a numerical forecast model: a comparison with observations. J. Appl. Meteorol. Climatol..

[20] Ouazzani ZR, Hacker JM, Thompson R, Peacock T (2014). The morning glory: flow visualization by mother nature. Phys. Fluids.

[21] Polyanin AD, Zaitsev VF (2003). Handbook of Exact Solutions for Ordinary Differential Equations.

[22] Porter A, Smyth NF (2002). Modelling the morning glory of the Gulf of Carpentaria. J. Fluid Mech..

[23] Robinson FJ, Patterson MD, Sherwood SC (2013). A numerical modeling study of the propagation of idealized sea-breeze density currents. J. Atmos. Sci..

[24] Rottman JW, Simpson JE (1989). Initiation and propagation of an atmospheric bore in a numerical forecast model: a comparison with observations. Q. J. R. Meteorol. Soc..

[25] Villari G (2016). A survival kit in phase plane analysis: some basic models and problems. Nonlinear Water Waves. Lecture Notes in Math.

